# Impact of multimodal education management on postoperative rehabilitation after total knee arthroplasty: A machine learning-based prediction model study

**DOI:** 10.1097/MD.0000000000047641

**Published:** 2026-02-13

**Authors:** Jingrong Wu, Jiayu Qian, Qiu Qian, Yu Gong, Jingyi Qian, Shuangyuan Du, Xiaojin Zhang, Lihong Xu

**Affiliations:** a Department of Orthopaedic Surgery, Zhangjiagang Hospital Affiliated to Soochow University (Zhangjiagang First People’s Hospital), Zhangjiagang, China; b Emergency Intensive Care Unit, Zhangjiagang Hospital Affiliated to Soochow University (Zhangjiagang First People’s Hospital), Zhangjiagang, China.

**Keywords:** illustrated pathway, interpretability analysis, machine learning, total knee arthroplasty, video education

## Abstract

This study aimed to evaluate the impact of multimodal education management using illustrated pathway with video education (IPVE) on rehabilitation quality after total knee arthroplasty. A retrospective cohort study design was adopted. Patients were grouped based on the median of final SF-36 quality of life scores. LASSO regression was used to screen predictive variables, and multivariate logistic regression was used to construct prediction models. The predictive performance of 5 machine learning algorithms was compared, and model efficacy was evaluated using ROC curves, calibration curves, and decision curve analysis. SHAP method was used to analyze feature importance. Multimodal education management using IPVE was significantly associated with better rehabilitation quality after total knee arthroplasty. A total of 223 patients who underwent total knee arthroplasty from October 2022 to January 2025 were included, with 121 cases (54.3%) in the high-quality rehabilitation group and 102 cases (45.7%) in the low-quality group. LASSO regression identified 4 key predictive variables: age, IPVE implementation, knee range of motion at discharge, and final knee function score. Multivariate logistic regression analysis showed that each 1-year increase in age reduced the probability of high-quality rehabilitation by 17.2% (*P*<.001), IPVE implementation was significantly associated with better rehabilitation quality (*P*<.001), each 1° increase in knee range of motion at discharge increased the probability of high-quality rehabilitation by 17.3% (*P*<.001), and each 1-point increase in final knee function score increased the probability of high-quality rehabilitation by 11.2% (*P* = .043). The random forest model performed best, with the AUC, sensitivity, specificity, accuracy, and F1 score all reaching 1.000, whereas the traditional logistic regression model had an AUC of 0.924. SHAP analysis showed that age was the most important predictive feature, and implementation of IPVE had a significant impact on rehabilitation quality. Multimodal educational management using an illustrated pathway combined with video-based education was significantly associated with improved rehabilitation quality after total knee arthroplasty.

## 1. Introduction

Total knee arthroplasty (TKA), as the “gold standard” for treating end-stage knee diseases, has become one of the most common elective orthopedic surgeries.^[1,2]^ With the intensifying population aging, the annual volume of TKA surgeries is rapidly increasing.^[3]^ However, postoperative rehabilitation outcomes show significant individual variations, with some patients experiencing poor functional recovery and limited quality of life improvement, which seriously affects overall surgical outcomes and patient satisfaction.^[4,5]^ Traditional TKA postoperative care predominantly adopts standardized and homogenized management models, lacking individualized interventions. Patients have insufficient rehabilitation knowledge and poor compliance, and problems such as fragmented health education content, single-format delivery, and absence of outcome evaluation are prominent.^[6]^ Studies have shown that systematic and individualized health education and nursing interventions can significantly improve rehabilitation outcomes, highlighting the urgent need to construct a diversified and systematic health education management model.^[7,8]^

Illustrated pathway with video education (IPVE), as an innovative multimodal health education management approach, integrates standardized care pathways, personalized illustrated educational materials, and multimedia video education to provide patients with visualized and easily understandable health education content, effectively improving disease awareness and rehabilitation knowledge mastery.^[9]^ International studies have shown that multimodal health interventions have demonstrated favorable effects in oncology,^[10]^ but their systematic application in orthopedics, particularly in postoperative rehabilitation management after TKA, remains in its early stages. Furthermore, accurately predicting postoperative rehabilitation risks, early identification of high-risk patients, and implementing precise interventions are key to improving rehabilitation quality.^[11,12]^ Traditional assessment relies on clinical experience and single indicators, lacking objectivity and accuracy. Machine learning technology, with its powerful data mining capabilities, has shown potential in medical prediction, but research on intelligent prediction models for TKA postoperative rehabilitation remains limited, and most models lack sufficient interpretability, which restricts clinical implementation.

Addressing these challenges, this study is the first to introduce the IPVE management model into postoperative nursing practice for TKA, constructing a systematic intervention program encompassing personalized illustrated education, video education, standardized care pathways, multidisciplinary collaboration, and continuous follow-up. Meanwhile, multiple machine learning algorithms were employed to construct postoperative rehabilitation quality prediction models, and SHAP (SHapley Additive exPlanations) interpretable artificial intelligence technology was adopted to enhance model transparency, providing scientific evidence for clinical decision-making. This study aims to: evaluate the impact of multimodal education management on postoperative rehabilitation quality after TKA; identify key risk factors affecting postoperative rehabilitation quality, and construct and validate machine learning-based rehabilitation risk prediction models; through SHAP interpretability analysis, deeply reveal the mechanisms by which each predictive factor influences rehabilitation outcomes, providing decision support for developing individualized clinical intervention strategies. Through this study, we expect to provide new theoretical foundations and practical paradigms for precision nursing management after TKA, promoting systematic improvement and intelligent development of perioperative nursing quality in orthopedics.

## 2. Methods

### 2.1. Study design and subject selection

This study adopted a cohort study design, selecting patients who underwent TKA in the Department of Orthopedic Surgery at Zhangjiagang Hospital Affiliated to Soochow University from October 2022 to January 2025 as research subjects. Inclusion criteria: patients aged 50 to 85 years undergoing TKA; meeting surgical indications for knee replacement, including degenerative osteoarthritis, inflammatory arthropathy, etc; receiving standardized postoperative rehabilitation treatment; complete clinical data; postoperative follow-up time ≥6 months to assess rehabilitation quality. Exclusion criteria: preexisting severe knee joint dysfunction affecting rehabilitation assessment; combined severe underlying diseases such as cardiopulmonary insufficiency, hepatorenal dysfunction, malignant tumors, etc; severe intraoperative or postoperative complications requiring reoperation; withdrawal from treatment or loss to follow-up; missing key clinical indicators exceeding 20%. This study was approved by the Institutional Review Board of Zhangjiagang Hospital Affiliated to Soochow University (approval number: ZJGYYLL-2025-07-007). The study was conducted in accordance with the Declaration of Helsinki. Written informed consent was obtained from all participants prior to enrollment.

### 2.2. Data collection

A uniformly designed case report form was used to collect patients’ clinical data, mainly including the following aspects: basic information (age, gender, BMI); preoperative assessment (preoperative self-care ability, visual analog scale [VAS] for pain, knee function score, knee range of motion [ROM], primary diagnosis, etc); surgery-related indicators (operation time, intraoperative blood loss, anesthesia method, and other perioperative indicators); postoperative interventions (implementation status of IPVE [yes vs no], time to first ambulation [≤24 hours vs 24–48 hours vs >48 hours], time to first oral intake [≤2 hours vs 2–4 hours vs >4 hours]); hospitalization monitoring indicators (postoperative hospital stay, VAS score at discharge, knee ROM at discharge, etc); and follow-up outcomes (final knee function score, final VAS score, final SF-36 quality of life score, etc).

### 2.3. Illustrated pathway with video education

**Figure 1.**
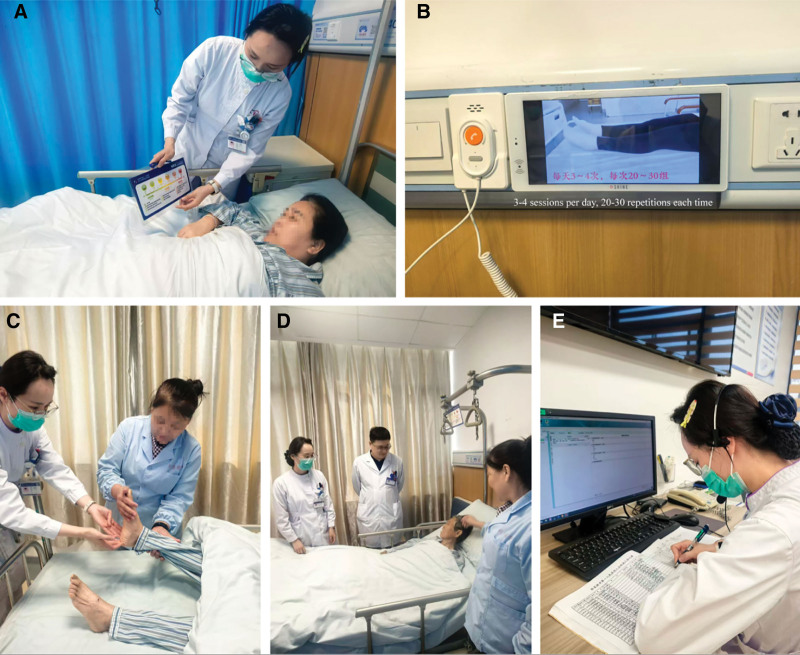
Implementation of the IPVE multimodal intervention model in TKA patients. (A) Personalized illustrated educational materials: Healthcare provider using tablet-based illustrated educational content with patient. (B) Educational video viewing: Standardized rehabilitation education video displayed on bedside monitor for patient viewing. (C) Standardized care pathway execution: Healthcare team implementing structured rehabilitation training and functional assessment. (D) Multidisciplinary team collaborative management: daily multidisciplinary rounds with physician-nurse-rehabilitation therapist collaboration. (E) Continuous communication and follow-up: healthcare providers conducting documentation and follow-up care coordination using electronic systems. IPVE = illustrated pathway with video education, TKA = total knee arthroplasty.

This study developed a standardized IPVE intervention model, including 5 core objectives (Fig. 1): Objective 1: Personalized illustrated educational materials. Standardized illustrated manuals were created, containing 4 modules: preoperative preparation, postoperative care, rehabilitation training, and complication prevention, using a combination of text and images, with each page equipped with standard diagrams and key explanations; Objective 2: Complete viewing of educational videos. A 25-minute standardized rehabilitation education video was produced, covering: preoperative preparation guidance (5 minutes), early postoperative rehabilitation training demonstration (10 minutes), home rehabilitation management (6 minutes), and frequently asked questions (4 minutes). Patients were required to watch completely and pass a comprehension test (≥8 points/10 points); Objective 3: Execution of standardized care pathway. Interventions were implemented according to time points: completion of health education assessment and individualized care plan upon admission; daily multidisciplinary rounds in the morning, rehabilitation training in the afternoon, and functional assessment in the evening from immediate postoperative period to discharge; completion of rehabilitation capacity assessment before discharge; Objective 4: Multidisciplinary team collaborative management. A physician-nurse-rehabilitation therapist-dietitian collaboration model was established, with daily morning rounds to discuss rehabilitation progress, weekly team meetings to set phase goals, and predischarge team assessment to formulate follow-up plans; Objective 5: Continuous communication and follow-up. A 24-hour nursing consultation hotline was provided, with standardized follow-up: telephone follow-up at 1 week, 1 month, and 3 months postdischarge, and outpatient follow-up at 1 month and 6 months.

IPVE assessment criteria: completion of ≥4 objectives was recorded as “yes,” <4 objectives was recorded as “no.” Quality control adopted quantitative assessment: illustrated material comprehension test ≥80%, video test ≥8 points, nursing intervention implementation ≥90%, team collaboration participation ≥80%, follow-up completion ≥80%.

### 2.4. Grouping

Using the median of SF-36 quality of life scores at final follow-up as the grouping criterion, patients were divided into 2 groups: patients with final SF-36 scores higher than or equal to the median were assigned to the good rehabilitation group, and patients with final SF-36 scores lower than the median were assigned to the poor group. The SF-36 scale is an internationally recognized quality of life assessment tool, containing 8 dimensions, with a total score of 0 to 100 points, where higher scores indicate better quality of life.^[13]^ This grouping method objectively reflects the overall postoperative rehabilitation outcomes of patients, providing a clear outcome variable definition for subsequent risk factor analysis and prediction model construction.

### 2.5. Statistical methods

R 4.2.2 and Python 3.9.6 software were used for statistical analysis. Continuous variables were tested for normality using the Shapiro–Wilk test. Normally distributed data were expressed as mean ± standard deviation (x̄±s), and intergroup comparisons were performed using independent sample t-tests; non-normally distributed data were expressed as median and interquartile range (M [Q1, Q3]), and intergroup comparisons were performed using Mann-Whitney U tests. Categorical variables were expressed as frequency and percentage (n [%]), and intergroup comparisons were performed using χ^2^ test or Fisher exact test. LASSO regression was used for variable screening, with 10-fold cross-validation to determine the optimal regularization parameter λ, and variables with nonzero coefficients under λ.1se were selected as candidate predictive factors; Pearson correlation analysis was used to test correlations between variables, with correlation coefficients <0.8 considered to indicate no severe multicollinearity.^[14]^ Multivariate logistic regression (LR) analysis was further performed on the screened variables using the backward method, calculating odds ratios and their 95% confidence intervals. Based on LR results, a rehabilitation risk prediction model was constructed, and a nomogram was drawn. The model formula was: Logit(P) = β_0_ + β_1_X_1_ + β_2_X_2_ +... + β_n_X_n_, where P is the probability of good rehabilitation, β_0_ is the intercept term, β is the regression coefficient, and X is the predictor variable. Model performance evaluation included discrimination (calculating AUC and its 95% confidence intervals through ROC curves, with AUC >0.7 considered to indicate good predictive ability), calibration (drawing calibration curves using Bootstrap resampling method), and clinical utility (decision curve analysis to evaluate net benefit at different thresholds).^[15]^ In addition, 5 machine learning algorithms were selected to compare predictive performance: LR as the traditional baseline method; random forest (RF) and extreme gradient boosting (XGB) as ensemble learning methods known for handling complex nonlinear relationships; support vector machine (SVM) for its effectiveness in high-dimensional classification; and naive Bayes (NB) as a probabilistic classifier with computational efficiency. This selection covered diverse algorithmic approaches to ensure comprehensive model comparison, with default parameter settings used to run each machine learning algorithm, and evaluation metrics including AUC, sensitivity, specificity, accuracy, and F1 score.^[16]^ To further enhance model interpretability, the SHAP method was used to analyze the optimal model, drawing feature importance plots and waterfall plots to quantify the contribution of each feature to the prediction results.^[17,18]^ All hypothesis tests were 2-sided, and *P* <.05 was considered statistically significant.

## 3. Results

### 3.1. Baseline patient characteristics

This study included 223 TKA patients, of which 102 cases (45.7%) were in the poor rehabilitation quality group and 121 cases (54.3%) were in the good group. There were no significant differences between the 2 groups in terms of gender, preoperative self-care ability, and primary diagnosis (*P* >.05). Patients in the poor group were significantly older than those in the good group (73.00 [70.00, 77.00] vs 67.00 [61.00, 70.00] years, *P* <.001), and had significantly lower preoperative knee function scores and ROM (*P* <.001). The proportion of patients receiving IPVE in the poor group was significantly lower than in the good group (41.18% vs 91.74%, *P* <.001), with longer postoperative hospital stays (*P* = .001). At discharge, knee ROM, final knee function score, and SF-36 score in the poor group were all significantly lower than those in the good group (all *P* <.001) (Table 1).

**Table 1:** Baseline characteristics of the high-quality and low-quality groups post-tkr rehabilitation.

Variables	Total (n = 223)	Poor group (n = 102)	Good group (n = 121)	Statistic	*P*
Age, M (Q1, Q3)	70.00 (65.00, 74.00)	73.00 (70.00, 77.00)	67.00 (61.00, 70.00)	Z = −7.351	<.001
Sex, n (%)					
Male	47 (21.08)	22 (21.57)	25 (20.66)	χ^2^=0.027	.869
Female	176 (78.92)	80 (78.43)	96 (79.34)		
BMI, M (Q1, Q3)	23.15 (21.54, 24.55)	22.18 (20.80, 23.78)	23.57 (21.69, 24.89)	Z = −3.462	<.001
Preop self-care, n (%)					
Independent	57 (25.56)	25 (24.51)	32 (26.45)	χ^2^=0.672	.714
Partially dependent	150 (67.26)	71 (69.61)	79 (65.29)		
Dependent	16 (7.17)	6 (5.88)	10 (8.26)		
Preop VAS, n (%)					
2	1 (0.45)	0 (0.00)	1 (0.83)	χ^2^=7.039	.134
3	72 (32.29)	37 (36.27)	35 (28.93)		
4	70 (31.39)	37 (36.27)	33 (27.27)		
5	58 (26.01)	21 (20.59)	37 (30.58)		
6	22 (9.87)	7 (6.86)	15 (12.40)		
Preop KFS, M (Q1, Q3)	53.00 (50.00, 55.00)	51.00 (49.00, 54.00)	54.00 (50.00, 56.00)	Z = −3.289	.001
Preop ROM, M (Q1, Q3)	91.00 (85.00, 96.00)	90.00 (85.00, 95.00)	95.00 (90.00, 99.00)	Z = −5.060	<.001
Primary diagnosis, n (%)					
Degenerative osteoarthritis	161 (72.20)	73 (71.57)	88 (72.73)	Fisher	.815
Inflammatory arthropathy	59 (26.46)	27 (26.47)	32 (26.45)		
Other knee disorders	3 (1.35)	2 (1.96)	1 (0.83)		
Operation time, M (Q1, Q3)	2.20 (2.00, 2.40)	2.10 (1.50, 2.30)	2.30 (2.00, 2.40)	Z = −2.840	.005
Intraop blood loss, M (Q1, Q3)	200.00 (150.00, 200.00)	200.00 (200.00, 200.00)	180.00 (150.00, 200.00)	Z = −4.823	<.001
Anesthesia method, n (%)					
General anesthesia with intubation	44 (19.73)	24 (23.53)	20 (16.53)	χ^2^=1.713	.191
Combined spinal-epidural anesthesia	179 (80.27)	78 (76.47)	101 (83.47)		
IPVE, n (%)					
No	70 (31.39)	60 (58.82)	10 (8.26)	χ^2^=65.690	<.001
Yes	153 (68.61)	42 (41.18)	111 (91.74)		
Postop hospital stay, M (Q1, Q3)	8.00 (6.00, 11.00)	9.00 (7.00, 11.00)	7.00 (6.00, 10.00)	Z = −3.222	.001
TFA, n (%)					
≤24h	153 (68.61)	60 (58.82)	93 (76.86)	Fisher	.012
24–48h	60 (26.91)	37 (36.27)	23 (19.01)		
>48h	10 (4.48)	5 (4.90)	5 (4.13)		
TFOI, n (%)					
≤2h	185 (82.96)	86 (84.31)	99 (81.82)	χ^2^=0.449	.799
2–4h	27 (12.11)	12 (11.76)	15 (12.40)		
>4h	11 (4.93)	4 (3.92)	7 (5.79)		
VAS at discharge, n (%)					
1	8 (3.59)	4 (3.92)	4 (3.31)	Fisher	.254
2	61 (27.35)	33 (32.35)	28 (23.14)		
3	136 (60.99)	55 (53.92)	81 (66.94)		
4	18 (8.07)	10 (9.80)	8 (6.61)		
Knee ROM at discharge, M (Q1, Q3)	103.00 (97.00, 109.50)	100.00 (95.00, 105.00)	105.00 (101.00, 112.00)	Z = −6.388	<.001
Final KFS, M (Q1, Q3)	66.00 (62.00, 69.00)	64.00 (60.00, 66.75)	68.00 (63.00, 70.00)	Z = −5.416	<.001
Final VAS, n (%)					
0	21 (9.42)	11 (10.78)	10 (8.26)	χ^2^=3.349	.341
1	72 (32.29)	37 (36.27)	35 (28.93)		
2	100 (44.84)	39 (38.24)	61 (50.41)		
3	30 (13.45)	15 (14.71)	15 (12.40)		
Final SF-36, M (Q_1_, Q_3_)	70.00 (65.00, 77.00)	64.00 (60.00, 68.00)	76.00 (72.00, 80.00)	Z = −12.869	<.001

Final KFS = final knee function score, Intraop Blood Loss = intraoperative blood loss, IPVE = illustrated pathway with video education, Preop KFS = preoperative knee function score, Preop Self-care = preoperative self-care ability, Preop ROM = preoperative knee ROM, Preop VAS = preoperative VAS, Postop Hospital Stay = postoperative hospital stay, ROM = range of motion, SF-36 = short form-36 health survey, TFA = time to first ambulation, TFOI = time to first oral intake, VAS = visual analog scale.

### 3.2. Predictive variable selection and regression model construction

**Figure 2.**
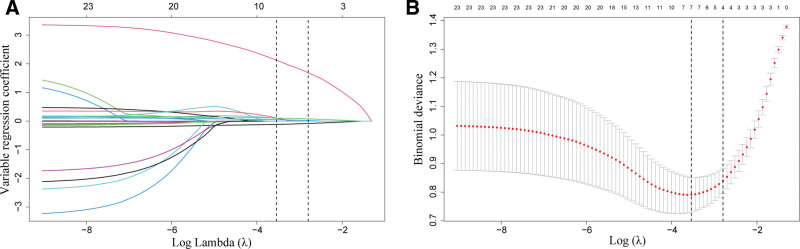
Results of risk factor selection for postoperative rehabilitation after TKA based on lasso regression. (A) LASSO coefficient profiles showing variable trajectories as Log λ increases, (B) cross-validation curve for optimal λ selection; the left dashed line indicates λ.min and the right dashed line indicates λ.1se. LASSO = least absolute shrinkage and selection operator, TKA = total knee arthroplasty.

**Figure 3.**
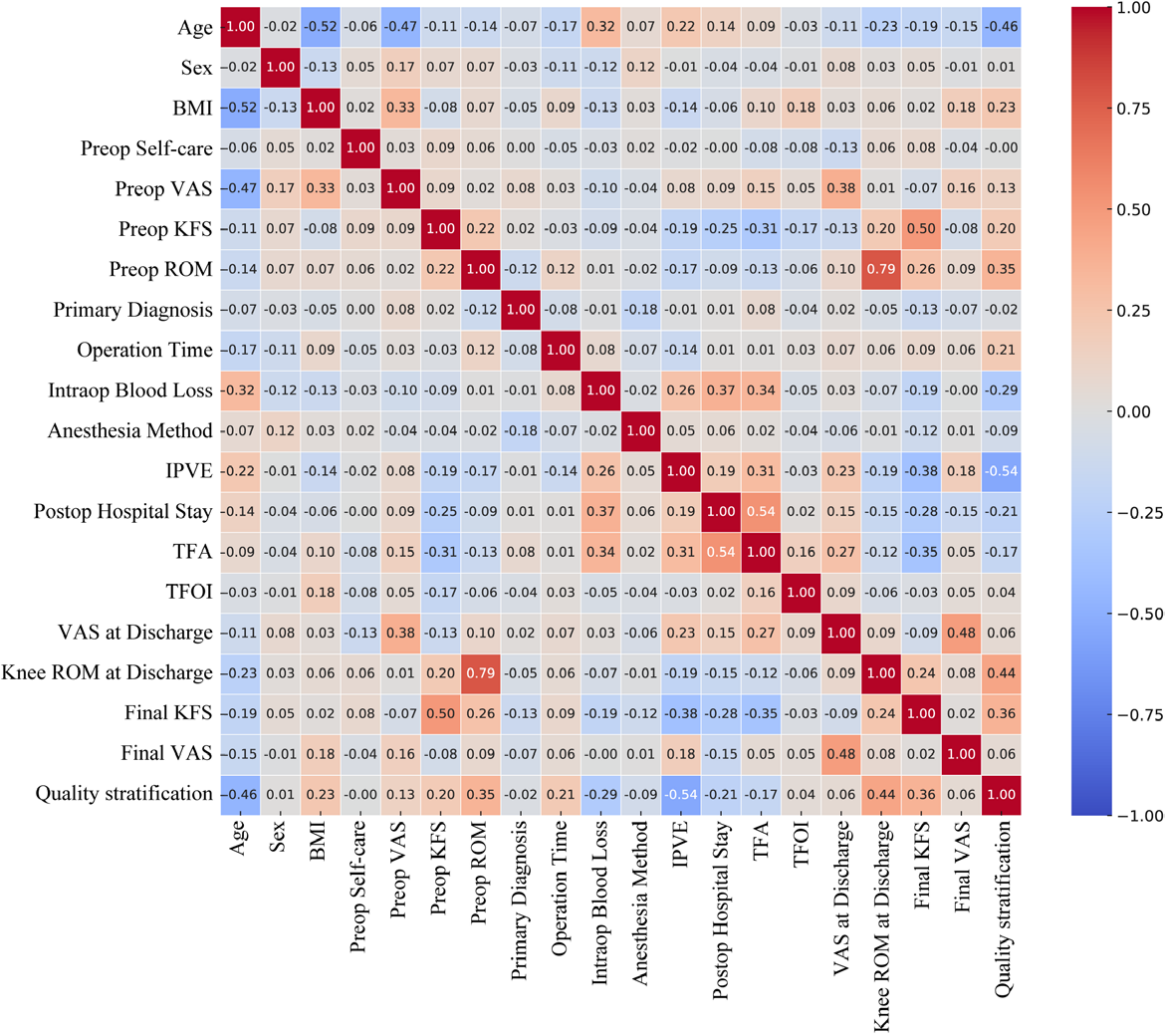
Heatmap of correlation analysis among risk prediction variables for postoperative rehabilitation after TKA. TKA = total knee arthroplasty.

LASSO regression was used to screen variables related to postoperative rehabilitation after TKA. Through 10-fold cross-validation to determine the optimal λ value, 4 key predictive variables were identified: age, IPVE, knee ROM at discharge, and final knee function score (Fig. 2). Pearson correlation analysis showed that correlation coefficients among all predictive variables were <0.8, indicating no severe multicollinearity problem and demonstrating the rationality of variable selection (Fig. 3). The 4 screened variables were included in multivariate LR analysis, and the results showed: each 1-year increase in age reduced the probability of good rehabilitation by 17.2% (OR = 0.828, 95% CI: 0.766–0.885, *P* <.001); IPVE implementation was significantly associated with improved rehabilitation quality (OR = 2.247, 95%CI: 1.381–4.371, *P* <.001); each 1° increase in knee ROM at discharge increased the probability of good rehabilitation by 17.3% (OR = 1.173, 95%CI: 1.102–1.259, *P* <.001); each 1-point increase in final knee function score increased the probability of good rehabilitation by 11.2% (OR = 1.112, 95% CI: 1.004–1.237, *P* = .043) (Table 2). The LR equation model was: Logit(P) = 2.897 × IPVE + 0.160 × knee ROM at discharge + 0.107 × final knee function score −0.189 × age −12.127, with binary variable coding: IPVE = yes entered as 1, IPVE = no entered as 0.

**Table 2:** Multivariate logistic regression analysis of risk predictive factors for rehabilitation after TKA.

Variables	β	SE	Wald χ^2^	*P*	OR (95% CI)
Age	−0.189	0.0364	26.905	<.001	0.828 (0.766–0.885)
IPVE-yes	2.897	0.51942	31.107	<.001	2.247 (1.381–4.371)
Knee ROM at discharge	0.16	0.03363	22.673	<.001	1.173 (1.102–1.259)
Final KFS	0.107	0.05288	4.080	.043	1.112 (1.004–1.237)

CI = confidence interval, Final KFS = final knee function score, IPVE = illustrated pathway with video education, OR = odds ratio, ROM = range of motion, TKA = total knee arthroplasty.

### 3.3. Construction of postoperative rehabilitation risk prediction model and nomogram

**Figure 4.**
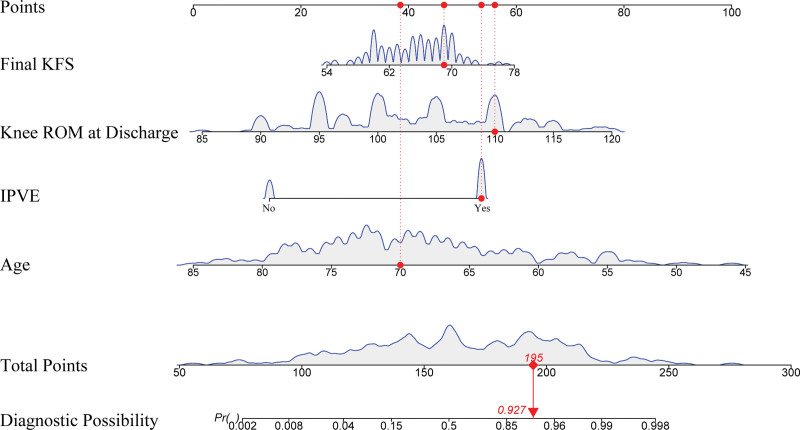
Nomogram of the risk prediction model for po.stoperative rehabilitation after TKA. TKA = total knee arthroplasty.

Based on the multivariate LR analysis results, a postoperative rehabilitation risk prediction model for TKA was constructed, and a nomogram was drawn for visual presentation (Fig. 4). In the nomogram, each predictive variable corresponds to a scoring axis. By finding the corresponding value on each variable axis and connecting vertically upward to the point axis, the score for that variable can be obtained. Adding all variable scores yields the total score, which is then projected vertically downward to the probability of good rehabilitation axis to obtain the predicted probability of good postoperative rehabilitation for that patient. The nomogram shows that age has the greatest impact on prediction results, with the widest scoring range; whether IPVE is implemented has a significant impact on rehabilitation quality; knee ROM at discharge and final knee function score are also important predictive factors.

### 3.4. Model performance evaluation and clinical utility assessment

**Figure 5.**
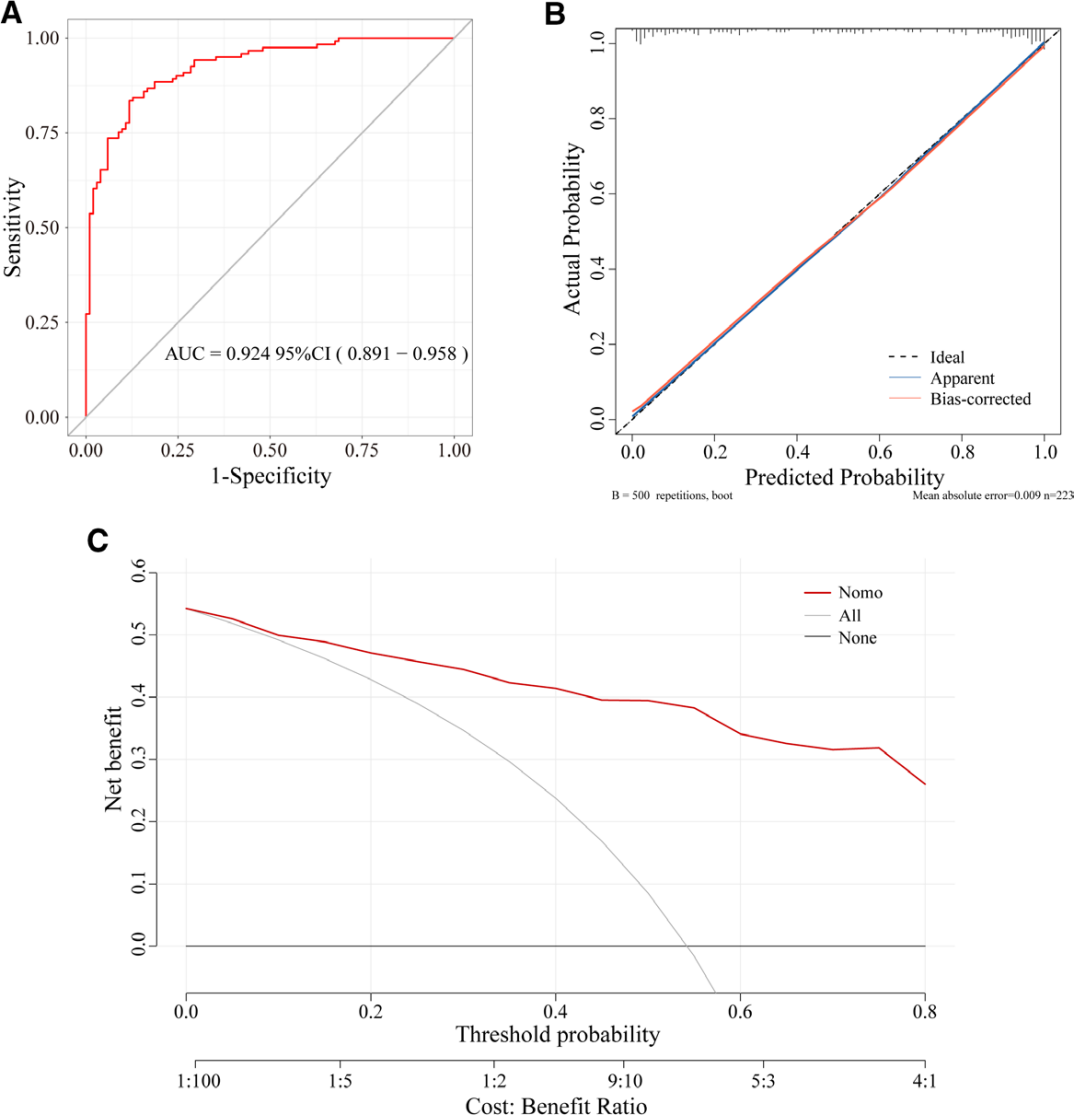
Performance evaluation of the risk prediction model for postoperative rehabilitation after TKA. (A) ROC curve showing AUC of 0.924 (95% CI: 0.891–0.958), (B) calibration curve with 500 bootstrap resamples showing mean absolute error of 0.009, (C) decision curve analysis demonstrating net benefit across threshold probabilities. AUC = area under the curve, CI = confidence interval, ROC = receiver operating characteristic, TKA = total knee arthroplasty.

Model performance evaluation showed that the prediction model had good discriminative ability, with an area under the ROC curve (AUC) of 0.924 (95%CI: 0.891–0.958), indicating that the model had good ability to identify patients with good and poor rehabilitation (Fig. 5A). Bootstrap calibration curve analysis showed that the model’s predicted probability was in good agreement with the actual occurrence probability, with a mean absolute error of 0.009, demonstrating high calibration (Fig. 5B). Decision curve analysis showed that within the threshold probability range of 0.1 to 0.8, this prediction model had higher net benefit than “treat all” and “treat none” strategies, demonstrating the clinical utility value of the model (Fig. 5C).

### 3.5. Performance comparison of multiple machine learning algorithms

**Figure 6.**
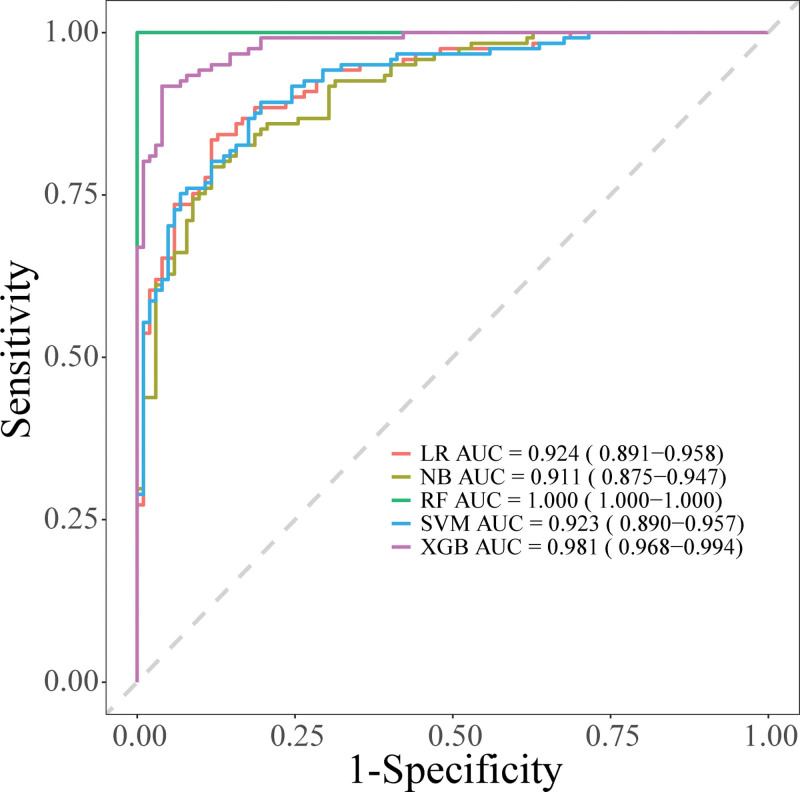
ROC curve evaluation of the discriminative performance of each machine learning model. ROC = receiver operating characteristic.

To validate the model’s effectiveness, the predictive performance of 5 machine learning algorithms was compared. Table 3 shows the performance indicators of 5 different machine learning models: the RF model performed best, with AUC, sensitivity, specificity, accuracy, and F1 score all reaching 1.000; the XGB model ranked second, with an AUC of 0.981 and accuracy of 0.965; the LR model had an AUC of 0.924 and accuracy of 0.894; the NB model had an AUC of 0.911 and accuracy of 0.889; the SVM model had relatively poor performance, with an AUC of 0.923 but accuracy of only 0.137. ROC curve analysis further validated the discriminative performance of each model (Fig. 6). The ROC curves of RF and XGB models were closest to the upper left corner, showing excellent discriminative ability. LR and NB models also demonstrated good predictive performance, while the SVM model’s ROC curve was relatively close to the diagonal line. Results indicated that machine learning-based prediction models all had good predictive performance, with the RF model performing most prominently.

**Table 3:** Performance evaluation of different machine learning models.

Prediction model	AUC	Sensitivity	Specificity	Accuracy	F1 score
LR model	0.924	0.835	0.882	0.894	0.863
RF model	1.000	1.000	1.000	1.000	1.000
XGB model	0.981	0.917	0.961	0.965	0.941
SVM model	0.923	0.107	0.196	0.137	0.120
NB model	0.911	0.793	0.882	0.889	0.838

LR = logistic regression, NB = naive Bayes, RF = random forest, SVM = support vector machine, XGB = extreme gradient boosting.

### 3.6. SHAP feature importance and interpretability analysis

**Figure 7.**
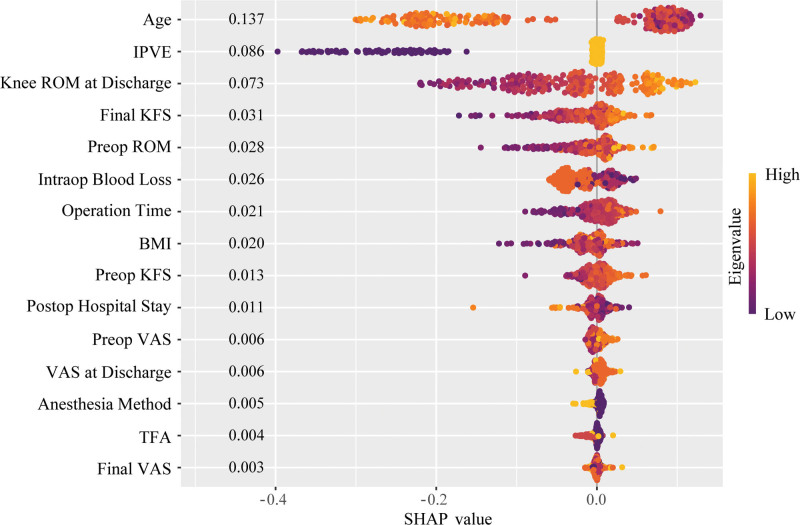
SHAP feature importance analysis of the risk prediction model for postoperative rehabilitation after TKA. SHAP = shapley additive explanations, TKA = total knee arthroplasty.

**Figure 8.**
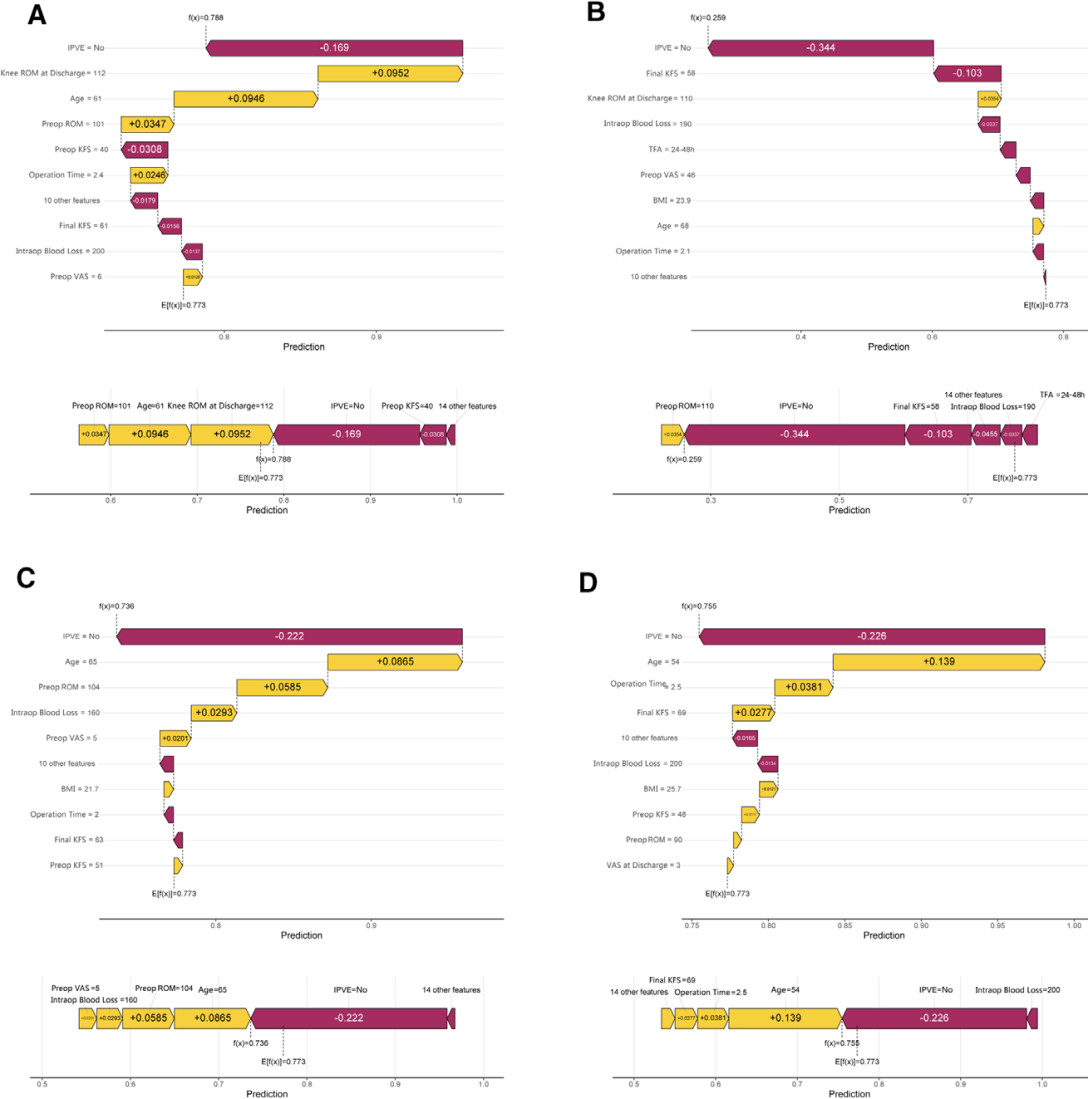
SHAP waterfall plot revealing key feature contributions in a typical case. (A–D) Four representative patient cases showing how each feature contributes to individual predictions. Yellow bars indicate features increasing the probability of good rehabilitation, while red bars indicate features decreasing this probability. SHAP = shapley additive explanations.

To deeply understand the model prediction mechanism and improve model interpretability, the SHAP method was used to perform feature importance analysis on the RF model. SHAP feature importance analysis showed that there were significant differences in the contribution of each predictive variable to the model output (Fig. 7). Age was the most important factor affecting postoperative rehabilitation quality after TKA, with the widest SHAP value distribution range and the most significant impact on prediction results. IPVE ranked second, with its implementation status having an important impact on rehabilitation quality prediction. Knee ROM at discharge and final knee function score were also important predictive factors, but with relatively smaller impact. SHAP waterfall plots further demonstrated the specific contributions of each feature to prediction results in typical cases (Fig. 8). The figure shows the process of how each feature progressively drives the change in final predicted probability starting from the baseline probability. Yellow bars represent factors that increase the probability of good rehabilitation, while red bars represent factors that decrease the probability.

## 4. Discussion

The multimodal education management of IPVE was significantly associated with better postoperative rehabilitation quality after TKA. The rate of good rehabilitation in patients receiving IPVE management reached 91.74%, while those not receiving it was only 8.26% (*P* <.001). Multivariate regression analysis further confirmed that IPVE implementation was significantly associated with improved rehabilitation quality. The significant association between multimodal education management and better outcomes may be explained by the following mechanisms: First, the combination of personalized illustrated educational materials and video education fully leverages the synergistic effects of multiple sensory channels such as visual and auditory pathways,^[19]^ effectively enhancing patients’ understanding and retention of rehabilitation knowledge. Studies have shown that multisensory learning can activate different brain regions, significantly improving information processing and retention effects.^[20]^ Second, standardized care pathways ensure the systematicity and continuity of rehabilitation interventions, avoiding the fragmentation problems common in traditional nursing care. Third, the multidisciplinary collaborative management model integrates professional resources including nurses, physicians, and rehabilitation therapists, providing patients with comprehensive and specialized rehabilitation guidance.^[21]^ Additionally, 24-hour nursing consultation and regular follow-up established a continuous support system, effectively improving patients’ rehabilitation compliance. Compared to traditional single-mode health education, IPVE’s multimodal characteristics can better adapt to different patients’ learning preferences and cognitive characteristics, which is particularly significant for elderly TKA patient populations. This provides important evidence for constructing precision-based and individualized postoperative care models.

Among the 4 key predictive factors identified through LASSO regression and multivariate LR analysis in this study, age, as the most important predictive factor, reduced the probability of good rehabilitation by 17.2% for each 1-year increase. This reflects the profound impact of age-related physiological changes on postoperative rehabilitation. With increasing age, patients’ muscle strength, bone density, soft tissue elasticity, and neuromuscular coordination all show varying degrees of deterioration, often accompanied by chronic diseases, decreased metabolic capacity, and weakened wound healing and tissue repair abilities.^[22]^ IPVE multimodal education management showed a strong association with rehabilitation outcomes, fully illustrating the core position of individualized and systematic health education in improving rehabilitation quality. Ho et al^[8]^ randomized controlled trial similarly confirmed this view, showing that structured health education can significantly improve patients’ rehabilitation compliance and functional recovery outcomes. Through multisensory channel synergy, IPVE was associated with better patient understanding and retention of rehabilitation knowledge but also establishes a continuous support system, effectively solving the information fragmentation problem in traditional education models. Each 1° increase in knee ROM at discharge was associated with an increased probability of good rehabilitation by 17.3%, reflecting the positive impact of early functional recovery status on long-term prognosis. This finding is consistent with Ma et al^[23]^ research conclusions on early postoperative joint ROM predicting long-term functional outcomes. Good early ROM not only reflects the success of surgical technique and tissue healing quality but also creates favorable conditions for subsequent rehabilitation training, effectively preventing joint stiffness and muscle atrophy.^[24]^ Although the final knee function score had relatively smaller predictive strength, each 1-point increase could still improve the probability of good rehabilitation by 11.2%, indicating the important value of functional assessment in comprehensive rehabilitation monitoring. It is worth noting that although factors such as BMI and preoperative knee function scores showed statistical differences in univariate analysis, they were not ultimately included in the multivariate analysis model, which may be related to the relatively lower predictive weight of these factors and their correlation with other variables.

Compared to traditional statistical methods, the machine learning algorithms introduced in this study demonstrate significant advantages in handling complex clinical data. Traditional LR analysis, although widely used in clinical prediction modeling and having good interpretability, has inherent limitations such as assuming linear relationships between variables and difficulty capturing complex nonlinear interactions.^[25]^ In contrast, machine learning algorithms can automatically identify hidden patterns in data and complex relationships between variables, demonstrating excellent learning capabilities when processing multidimensional clinical data.^[26]^ Among the 5 prediction models in this study, the RF model performed most prominently, with AUC, sensitivity, specificity, accuracy, and F1 score all achieving perfect prediction results of 1.000, significantly outperforming the traditional LR model (AUC = 0.924, accuracy = 0.894). It is noteworthy that RF, as an ensemble learning algorithm, can effectively handle nonlinear relationships between features through mechanisms of constructing multiple decision trees and comprehensive voting, while possessing good anti-overfitting ability and generalization performance. The XGB algorithm (XGBoost) also performed excellently, with AUC reaching 0.981 and accuracy of 0.965, validating the technical advantages of tree-based ensemble models in handling postoperative rehabilitation prediction problems after TKA. In contrast, although SVMs theoretically possess strong nonlinear mapping capabilities, they performed relatively poorly in this study, with an AUC of 0.923 but accuracy of only 0.137, which may be related to factors such as high-dimensional small sample data characteristics, kernel function selection, and improper parameter tuning. The NB model, based on feature independence assumptions, has certain limitations when processing clinical data with feature correlations, but still achieved an AUC value of 0.911, indicating its applicability in this application scenario. The introduction of machine learning methods provides more precise and individualized prediction tools for clinical decision-making.

The introduction of SHAP interpretability analysis technology effectively addresses the clinical application barriers caused by the “black box” characteristics of machine learning models.^[27]^ Although machine learning algorithms have excellent predictive performance, their complex internal computational processes often lack transparency, making it difficult for clinical healthcare personnel to understand the model’s decision logic, which restricts their widespread application in clinical practice.^[28]^ The SHAP method, based on the Shapley value concept in game theory, can quantify the specific contribution of each feature to model prediction results, achieving organic unity of high-precision prediction and model interpretability. In this study, SHAP feature importance analysis revealed the internal mechanisms of postoperative rehabilitation quality prediction after TKA. Results showed that age, as the most critical predictive factor, had the widest SHAP value distribution range and the most significant impact on model output, which is highly consistent with clinical observations of the negative impact of age-related physiological function decline on rehabilitation outcomes. IPVE multimodal education management ranked second, and its predictive value further confirmed the important role of structured health education in improving patient rehabilitation quality. Although knee ROM at discharge and final knee function score had relatively lower importance, they still made positive contributions to prediction results, reflecting the predictive value of early functional recovery status and sustained functional improvement for long-term rehabilitation quality. The visual presentation of SHAP waterfall plots provides intuitive tools for individualized clinical decision-making. By displaying the stepwise contribution process of each feature from baseline probability to final predicted probability in typical cases, clinical healthcare personnel can clearly understand each patient’s risk composition, thereby formulating targeted intervention strategies.

This study has certain limitations. First, as a single-center retrospective study, the sample source is limited, selection bias may exist, and external validity needs further verification. Second, although the sample size meets statistical requirements, it is still insufficient for machine learning model training, which may affect generalization ability. Third, the follow-up time is relatively short, making it difficult to comprehensively evaluate long-term rehabilitation outcomes and complications. Finally, given the retrospective observational design, causal relationships between IPVE implementation and rehabilitation outcomes cannot be established due to potential unmeasured confounding. Future plans include conducting multicenter prospective studies, expanding sample size and extending follow-up to 1 to 3 years, incorporating more predictive dimensions, constructing more comprehensive models, and developing decision support tools to achieve a paradigm shift from evidence-based nursing to precision nursing.

## 5. Conclusion

This study constructed a postoperative rehabilitation quality prediction model for TKA. Machine learning algorithms outperformed traditional statistical methods, demonstrating stronger predictive capabilities and advantages in handling complex clinical data. The multimodal education management model was significantly associated with better rehabilitation outcomes. This model helps with early identification of rehabilitation risks, supports personalized nursing decisions, and is of great significance for improving patient prognosis and optimizing rehabilitation resource allocation.

## Author contributions

**Conceptualization:** Jingrong Wu.

**Data curation:** Jiayu Qian.

**Funding acquisition:** Jingrong Wu.

**Investigation:** Jiayu Qian, Qiu Qian, Yu Gong, Jingyi Qian, Shuangyuan Du, Xiaojin Zhang.

**Resources:** Qiu Qian, Jingyi Qian.

**Software:** Qiu Qian, Jingyi Qian.

**Supervision:** Lihong Xu.

**Validation:** Jingyi Qian.

**Writing – original draft:** Jingrong Wu, Lihong Xu.

**Writing – review & editing:** Lihong Xu.
